# Influences of the Integrated Rice-Crayfish Farming System with Different Stocking Densities on the Paddy Soil Microbiomes

**DOI:** 10.3390/ijms25073786

**Published:** 2024-03-28

**Authors:** Yiran Hou, Rui Jia, Wei Sun, Bing Li, Jian Zhu

**Affiliations:** 1 Key Laboratory of Integrated Rice-Fish Farming Ecology, Ministry of Agriculture and Rural Affairs, Freshwater Fisheries Research Center, Chinese Academy of Fishery Sciences, Wuxi 214081, China; houyr@ffrc.cn (Y.H.); jiar@ffrc.cn (R.J.); sunwei_ffrc@hotmail.com (W.S.); 2 Wuxi Fisheries College, Nanjing Agricultural University, Wuxi 214081, China

**Keywords:** red claw crayfish, stocking density, paddy field, soil bacterial community, agricultural production patterns

## Abstract

Integrated rice-fish farming has emerged as a novel agricultural production pattern to address global food security challenges. Aiming to determine the optimal, scientifically sound, and sustainable stocking density of red claw crayfish (*Cherax quadricarinatus*) in an integrated rice-crayfish farming system, we employed Illumina high-throughput 16S rRNA gene sequencing to evaluate the impact of different stocking densities of red claw crayfish on the composition, diversity, function, and co-occurrence network patterns of soil bacterial communities. The high stocking density of red claw crayfish reduced the diversity and evenness of the soil bacterial community during the mid-culture stage. *Proteobacteria*, *Actinobacteria*, and *Chloroflexi* emerged as the most prevalent phyla throughout the experimental period. Low stocking densities initially boosted the relative abundance of Actinobacteria in the paddy soil, while high densities did so during the middle and final stages. There were 90 distinct functional groups identified across all the paddy soil samples, with chemoheterotrophy and aerobic chemoheterotrophy being the most abundant. Low stocking densities initially favored these functional groups, whereas high densities enhanced their relative abundances in the later stages of cultivation. Medium stocking density of red claw crayfish led to a more complex bacterial community during the mid- and final culture stages. The experimental period showed significant correlations with soil bacterial communities, with total nitrogen (TN) and total phosphorus (TP) concentrations emerging as primary factors contributing to the alterations in soil bacterial communities. In summary, our findings demonstrated that integrated rice-crayfish farming significantly impacted the soil microbiomes and environmental factors at varying stocking densities. Our study contributed to theoretical insights into the profound impact of integrated rice-crayfish farming with various stocking densities on bacterial communities in paddy soils.

## 1. Introduction

With the ongoing global population increase, food security has emerged as a critical worldwide challenge. Rice and aquatic products, as fundamental food sources, are pivotal to addressing this issue [1,2,3]. Recent advancements in agricultural production patterns, notably integrated rice-fish farming, have brought significant improvements in sustainable agricultural production and adequate food supply [4,5]. Integrated rice-fish farming, which merges aquaculture with rice cultivation, establishes mutually beneficial relationships between rice plants and aquatic animals to realize more stable agroecosystems, enhance resource utilization, and boost food production, thereby promoting agricultural productivity, safeguarding food security, and ensuring a reliable supply of high-quality protein [6,7,8,9,10]. By 2023, China’s integrated rice-fish farming areas had grown to encompass over 2.86 million hectares, making up 9.72% of the nation’s total rice cultivation area [4,5].

Integrated rice-fish farming incorporates various aquatic species, including red swamp crayfish *Procambarus clarkii*, red claw crayfish *Cherax quadricarinatus*, common carp, and river crab *Eriocheir sinensis* [11,12,13,14,15,16]. Of these, integrated rice-crayfish farming has been widely adopted in China, leading in both the scale of implementation and production output [4,5]. The red claw crayfish *C. quadricarinatus* is distinguished by its considerable size, swift growth rate, robust disease resistance, stronger adaptability, and significant economic value, making it an integral choice for integrated rice-crayfish farming since its introduction to China in 1992 [13,14,17,18,19]. As typical benthic organisms, crayfish can influence aquatic ecosystems, playing a crucial role in biogeochemical cycles and altering benthic bacterial communities at the sediment-water interface [20,21,22,23,24,25]. Meanwhile, exogenous artificial nutrition input in the integrated rice-fish farming system can also have significant impacts on the agroecosystem. However, studies on the ecological effects of integrated rice-crayfish farming practices, particularly research on establishing scientifically sound stocking densities of aquatic animals from the perspective of ecological effects, are still limited.

Bacterial communities in water, soils, or sediments play a pivotal role in agricultural productivity by facilitating migration and transformation of nutrients, enhancing soil fertility, maintaining ecological structure at the soil-water interface, supporting plant health, and protecting plants from abiotic stresses [26,27,28,29,30,31,32,33]. Additionally, bacterial communities serve as an important and integral indicator for assessing the variations in agroecosystems, offering insights into their ecological status [34,35]. In agroecosystems, different agricultural production patterns or different management approaches can induce several environmental changes, thus affecting the bacterial communities [9,36,37,38,39]. Hence, to comprehend how agroecosystems respond and adapt to the environmental changes induced by various agricultural production patterns, revealing the spatiotemporal variations in bacterial communities is crucial [40,41,42]. This understanding not only aids in grasping the dynamics of agroecosystems but also serves as a fundamental approach to establishing and optimizing sustainable agricultural production systems.

Therefore, in this study, we employed Illumina high-throughput 16S rRNA gene sequencing to thoroughly analyze and compare the soil bacterial communities influenced by integrated rice-crayfish farming across varying stocking densities throughout the experiment. We explored the effects of integrated rice-crayfish farming with different stocking densities on the composition, diversity, function, and co-occurrence network patterns of the soil bacterial communities. Additionally, we assessed the relationships between environmental variables and the soil bacterial communities. Our objective is to offer theoretical insights into the extensive impact of integrated rice-crayfish farming with varying stocking densities on agricultural ecosystems, aiming to guide the establishment of scientifically sound and sustainable agricultural practices. For the present study, we hypothesized that integrated rice-crayfish farming with different stocking densities would have obviously different impacts on bacterial communities within paddy soils and that changes in soil bacterial communities would be significantly correlated with variations in environmental factors.

## 2. Results

### 2.1. The Bacterial Community Diversity within Paddy Soil

**Figure 1:**
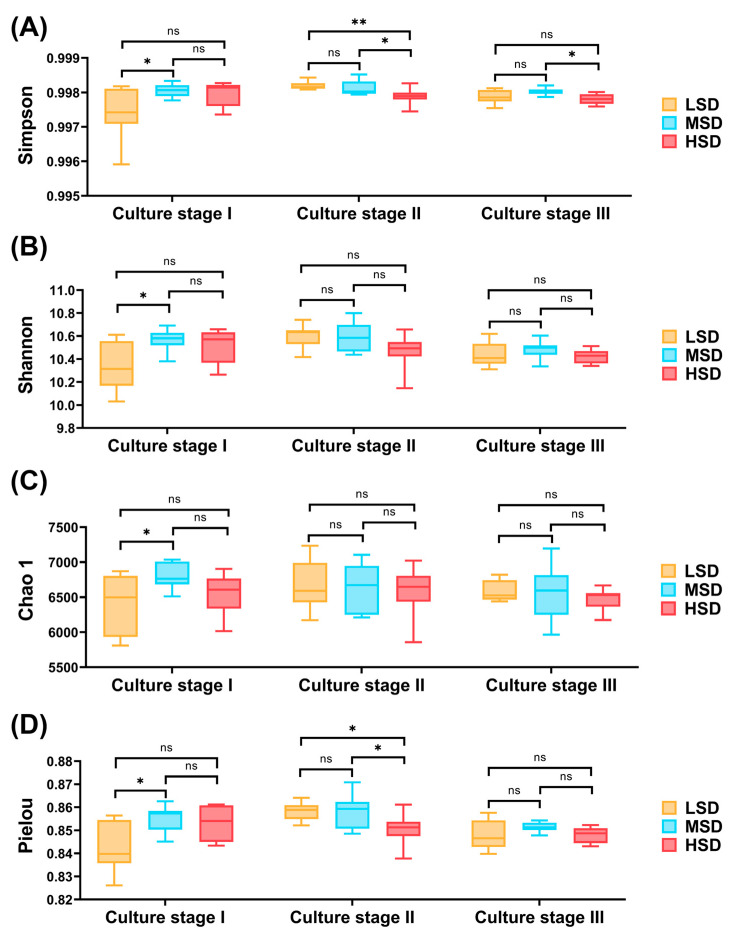
Alpha diversity of the bacterial community within paddy soil. (**A**) Differences in the Simpson index for the soil bacterial community between LSD, MSD, and HSD groups. (**B**) Differences in the Shannon index for the soil bacterial community between LSD, MSD, and HSD groups. (**C**) Differences in the Chao 1 index for the soil bacterial community between LSD, MSD, and HSD groups. (**D**) Differences in the Pielou_J index for the soil bacterial community between LSD, MSD, and HSD groups. “**”, “*”, and “ns” indicated extremely significant (*p* < 0.01), significant (*p* < 0.05), and nonsignificant differences (*p* > 0.05), respectively. The LSD, MSD, and HSD represented low, medium, and high stocking densities, respectively. The culture stages I, II, and III indicated that the paddy soil samples were collected on 30 August, 30 September, and 30 October, respectively.

Effects derived from the different stocking densities on the bacterial community diversity within the paddy soil are illustrated in Figure 1. During culture stage I, the LSD group demonstrated significantly lower values in the Simpson, Shannon, Chao 1, and Pielou_J indices compared to the MSD group (Figure 1, *p* < 0.05). However, no significant differences were observed in any diversity indices between the LSD and HSD groups, as well as between the MSD and HSD groups (Figure 1, *p* > 0.05). In culture stage II, the Simpson and Pielou_J indices for the HSD group were obviously lower than those for both the LSD and MSD groups (Figure 1, *p* < 0.05), while the Shannon and Chao1 indices showed no significant differences across the groups (Figure 1, *p* < 0.05). By culture stage III, the Shannon, Pielou_J, and Chao1 indices did not notably differ among the treatment groups (Figure 1, *p* > 0.05), except for the Simpson index, which was significantly lower in the HSD group compared to the MSD group (Figure 1, *p* < 0.05).

### 2.2. The Bacterial Community Composition within Paddy Soil

**Figure 2:**
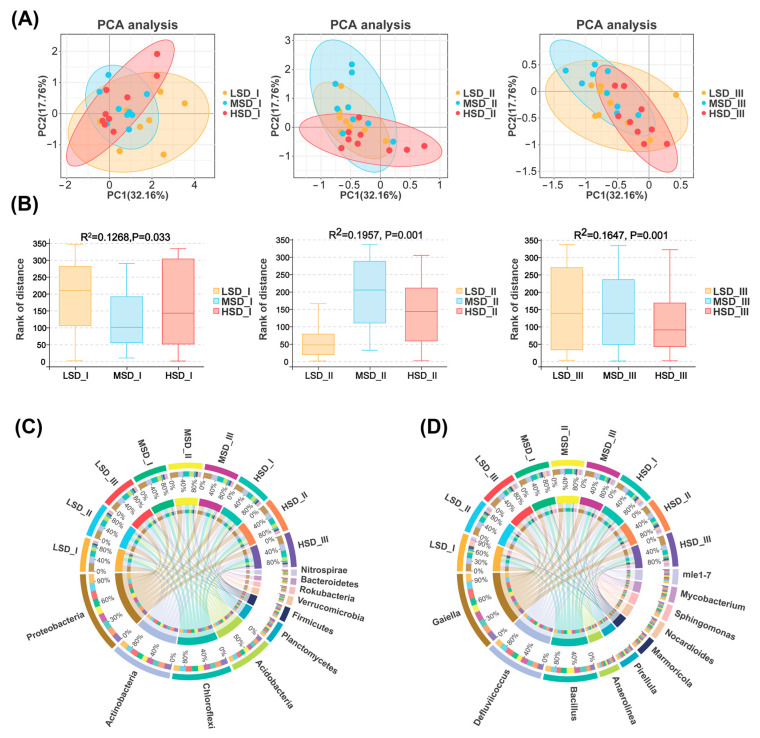
Differences in the bacterial community within paddy soil across the LSD, MSD, and HSD groups. (**A**) Principal component analysis (PCA) to evaluate the effects of varying stocking densities on the soil bacterial communities within integrated rice-crayfish farming systems. (**B**) Adonis tests for further statistically confirming the discrepancy presented by PCA. (**C**) The dominant bacterial phyla (top 10 in relative abundance) in the paddy soils during the entire experimental period. (**D**) The dominant bacterial genera, also ranked by relative abundance, were observed in the paddy soils throughout the experimental period. The LSD, MSD, and HSD denoted the low, medium, and high stocking densities, respectively, while the suffix symbols “I”, “II”, and “III” indicated the distinct sampling times corresponding to culture stages I (30 August), II (30 September), and III (30 October), respectively.

**Figure 3:**
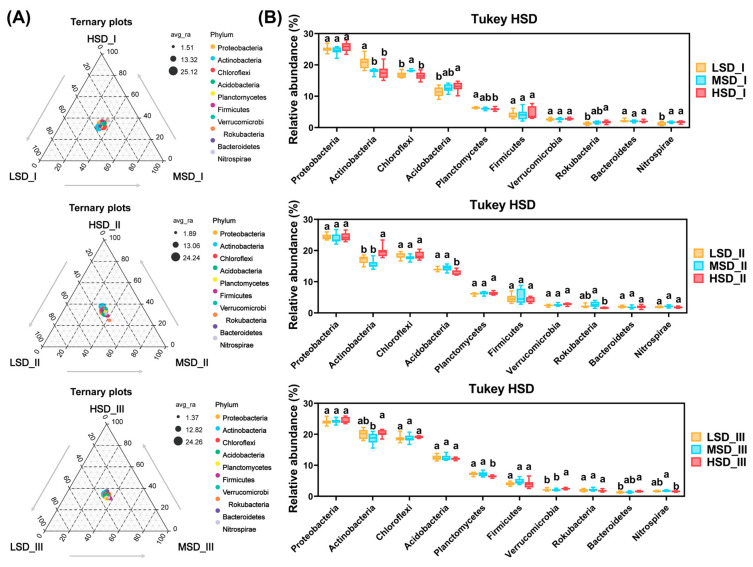
Differences in the ten most dominant bacterial phyla by relative abundance within paddy soils across the LSD, MSD, and HSD groups. (**A**) Ternary plot to visualize the differences in dominant bacterial phyla among the LSD, MSD, and HSD groups at each culture stage. (**B**) Tukey’s honestly significant difference (HSD) to statistically ascertain the discrepancies in the relative abundances of these dominant bacterial phyla across the LSD, MSD, and HSD groups at each culture stage. The abbreviations LSD, MSD, and HSD corresponded to the low, medium, and high stocking densities, respectively, while the symbols “I”, “II”, and “III” denoted the specific sampling times at culture stages I (30 August), II (30 September), and III (30 October), respectively. The notation of different lowercase letters signified significant differences (*p* < 0.05) among the groups.

**Figure 4:**
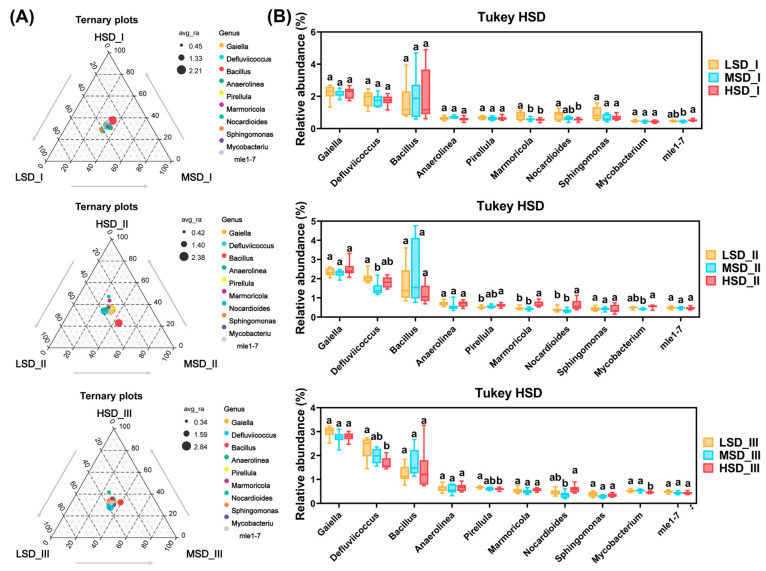
Differences in the ten most dominant bacterial genera by relative abundance within paddy soils across the LSD, MSD, and HSD groups. (**A**) Ternary plot to visualize the differences in dominant bacterial genera among the LSD, MSD, and HSD groups at each culture stage. (**B**) Tukey’s honestly significant difference (HSD) to statistically ascertain the discrepancies in the relative abundances of these dominant bacterial genera across the LSD, MSD, and HSD groups at each culture stage. The abbreviations LSD, MSD, and HSD corresponded to the low, medium, and high stocking densities, respectively, while the symbols “I”, “II”, and “III” denoted the specific sampling times at culture stages I (30 August), II (30 September), and III (30 October), respectively. The notation of different lowercase letters signified significant differences (*p* < 0.05) among the groups.

The impacts of the different stocking densities on the soil bacterial community composition within the integrated rice-crayfish farming system have been revealed in Figure 2, Figure 3 and Figure 4. The PCA, in conjunction with the Adonis test, highlighted significant disparities in the bacterial community composition across the LSD, MSD, and HSD groups at different culture stages. Throughout the experimental period, the top 10 most predominant bacterial phyla were *Proteobacteria*, *Actinobacteria*, *Chloroflexi*, *Acidobacteria*, *Planctomycetes*, *Firmicutes*, *Verrucomicrobia*, *Rokubacteria*, *Bacteroidetes*, and *Nitrospirae* (Figure 2C). The top 10 genera with the highest relative abundance were *Gaiella*, *Defluviicoccus*, *Bacillus*, *Anaerolinea*, *Pirellula*, *Marmoricola*, *Nocardioides*, *Sphingomonas*, *Mycobacterium*, and *mle 1–7* (Figure 2D).

The integrated rice-crayfish farming system with different stocking densities markedly influenced the relative abundances of the top 10 most abundant bacterial phyla and genera, as illustrated in Figure 3 and Figure 4. During culture stage I, the LSD group exhibited the markedly highest relative abundances of the phylum *Actinobacteria*, while the MSD group showed similar trends for the phylum *Chloroflexi* when compared to other groups (Figure 3B, *p* < 0.05). The HSD group had significantly greater relative abundances of *Acidobacteria* and *Rokubacteria* than the LSD group (Figure 3B, *p* < 0.05). Additionally, the relative abundance of *Nitrospirae* in the LSD group was notably lower than in other groups (Figure 3B, *p* < 0.05). In culture stage II, the HSD group showed a significantly higher relative abundance of Actinobacteria and a lower relative abundance of *Acidobacteria* compared to other groups (Figure 3B, *p* < 0.05). The HSD group also had a significantly lower relative abundance of *Rokubacteria* than the MSD group (Figure 3B, *p* < 0.05). By culture stage III, the HSD group demonstrated a significant decrease in the relative abundance of *Planctomycetes* and an increase in the relative abundance of *Verrucomicrobia* relative to other groups (Figure 3B, *p* < 0.05). Furthermore, the relative abundances of *Actinobacteria* and *Bacteroidetes* in the HSD group were significantly higher than those in the MSD and LSD groups, respectively, with *Nitrospirae* being significantly lower in the HSD group compared to the MSD group (Figure 3B, *p* < 0.05).

Regarding the top 10 most abundant bacterial genera, the LSD group had a significantly higher relative abundance of the genus *Marmoricola* at culture stage I compared to other groups (Figure 4B, *p* < 0.05). When it came to culture stage II, the HSD group maintained the highest relative abundance of *Marmoricola* (Figure 4B, *p* < 0.05). No significant differences were observed in the relative abundance of *Defluvlicoccus* among the LSD, MSD, and HSD groups at culture stage I (Figure 4B, *p* > 0.05). However, the LSD group exhibited the highest relative abundance of *Defluvlicoccus* at culture stages II and III, with significant differences noted against the MSD and HSD groups at each respective stage (Figure 4b, *p* < 0.05). The relative abundance of *Nocardioides* was lowest in the HSD group at culture stage I, but it was significantly higher in the HSD group compared to others at stages II, being notably higher than in the MSD group at the final culture stage (Figure 4B, *p* < 0.05).

### 2.3. Function Predictions for the Soil Bacterial Community

**Figure 5:**
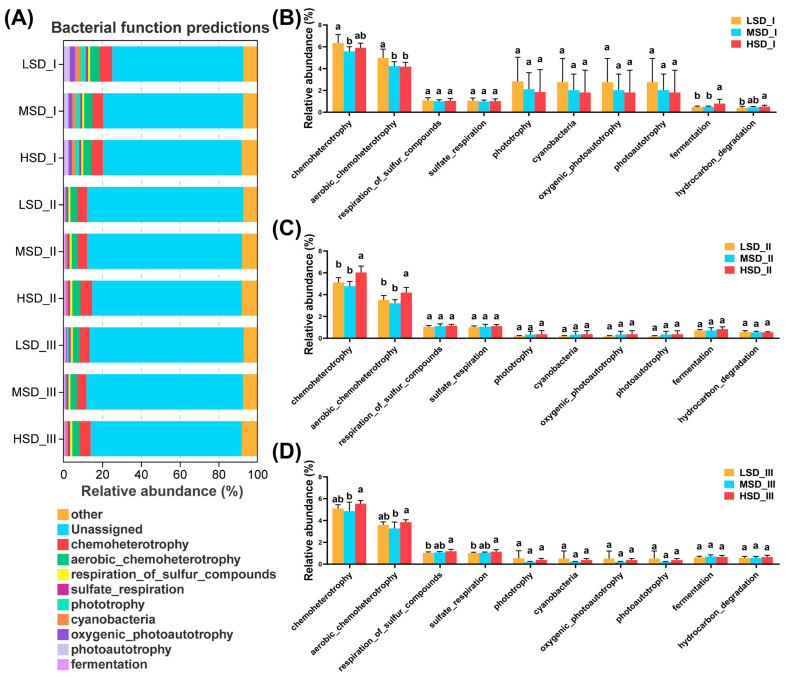
Function prediction for the bacterial community in paddy soil based on the Functional Annotation of Prokaryotic Taxa (FAPROTAX). (**A**) The dominant functional groups (top 10 in the relative abundance) for the soil bacterial communities during the experimental period. (**B**–**D**) Differences in the relative abundances of the dominant functional groups between the LSD, MSD, and HSD groups at each culture stage. The LSD, MSD, and HSD represented low, medium, and high stocking densities, respectively. The suffix symbols “I”, “II”, and “III” represented the different sampling times, namely culture stage I (30 August), culture stage II (30 September), and culture stage III (30 October), respectively. The notation of different lowercase letters signified significant differences (*p* < 0.05) among the groups.

In our study, we identified 90 distinct functional groups across all the paddy soil samples throughout the experiment, with chemoheterotrophy, aerobic chemoheterotrophy, respiration of sulfur compounds, sulfate respiration, phototrophy, cyanobacteria, oxygenic photoautotrophy, photoautotrophy, fermentation, and hydrocarbon degradation being the top 10 most abundant ranked by relative abundance (Figure 5A). Integrated rice-crayfish farming with varying stocking densities had a pronounced impact on the bacterial community functional groups within paddy soil (Figure 5B,C, *p* < 0.05). Specifically, during culture stage I, the LSD group exhibited significantly higher relative abundance of the chemoheterotrophy than the MSD group, with its aerobic chemoheterotrophy level also surpassing that of other groups (Figure 5B, *p* < 0.05). Additionally, the HSD group displayed significantly greater relative abundances of fermentation and hydrocarbon degradation compared to the other groups (Figure 5B, *p* < 0.05). During the second culture stage II, the HSD group had notably higher relative abundances of chemoheterotrophy and aerobic chemoheterotrophy than the other groups (Figure 5C, *p* < 0.05), with no significant differences in other functional groups across the LSD, MSD, and HSD groups (Figure 5C, *p* > 0.05). By culture stage III, the HSD group maintained the highest relative abundances of chemoheterotrophy and aerobic chemoheterotrophy, significantly exceeding the MSD group (Figure 5D, *p* < 0.05). Moreover, the HSD group also demonstrated the highest relative abundances of sulfur compound respiration and sulfate respiration, significantly outperforming the LSD group (Figure 5D, *p* < 0.05).

### 2.4. Co-Occurrence Network Patterns for the Soil Bacterial Community

**Figure 6:**
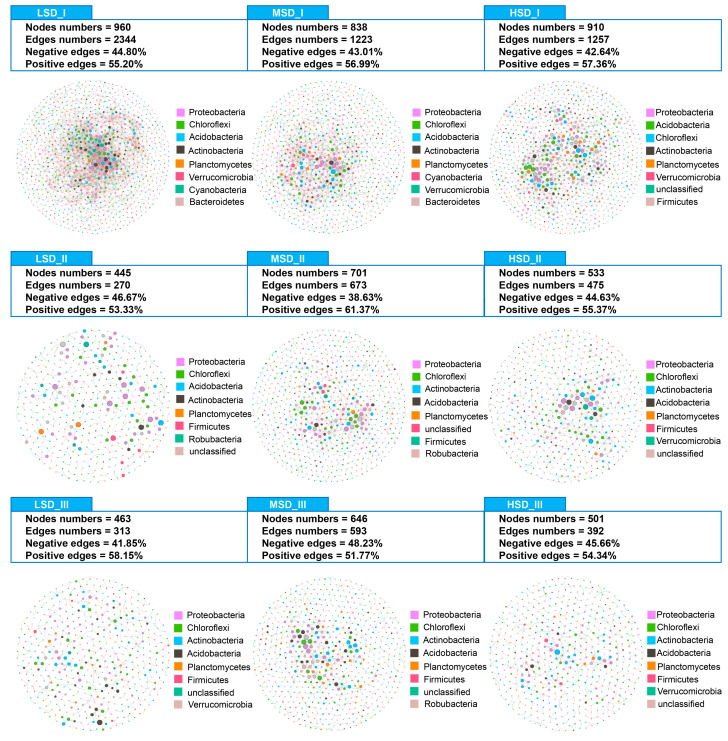
Co-occurrence networks for the bacterial communities in the paddy soil. The abbreviations LSD, MSD, and HSD corresponded to the low, medium, and high stocking densities, respectively, while the symbols “I”, “II”, and “III” denoted the specific sampling times at culture stages I (30 August), II (30 September), and III (30 October), respectively.

Co-occurrence networks for the soil bacterial communities have been constructed to investigate the soil bacterial patterns and are visualized in Figure 6. In culture stage I, the co-occurrence network for the LSD, MSD, and HSD groups featured 960, 838, and 910 nodes, with 2344, 1233, and 1257 edges, respectively (Figure 6). The proportion of negative to total edges in these networks was 44.80%, 43.01%, and 42.64% for the LSD, MSD, and HSD groups, respectively (Figure 6). During the culture stage II, the co-occurrence network for the soil bacterial community in the LSD, MSD, and HSD groups comprised 445 nodes and 270 edges, 701 nodes and 673 edges, and 533 nodes and 475 edges, respectively (Figure 6). For the LSD, MSD, and HSD groups, the negative to total edge ratios were 46.67%, 38.63%, and 44.63%, respectively (Figure 6). By culture stage III, the networks consisted of 463, 646, and 501 nodes with 313, 593, and 392 edges for the LSD, MSD, and HSD groups, respectively. The ratios of negative to total edges were 41.85%, 48.23%, and 45.66% for the LSD, MSD, and HSD groups, respectively.

### 2.5. Variations in the Environmental Factors and Their Relationships with the Soil Bacterial Community

**Figure 7:**
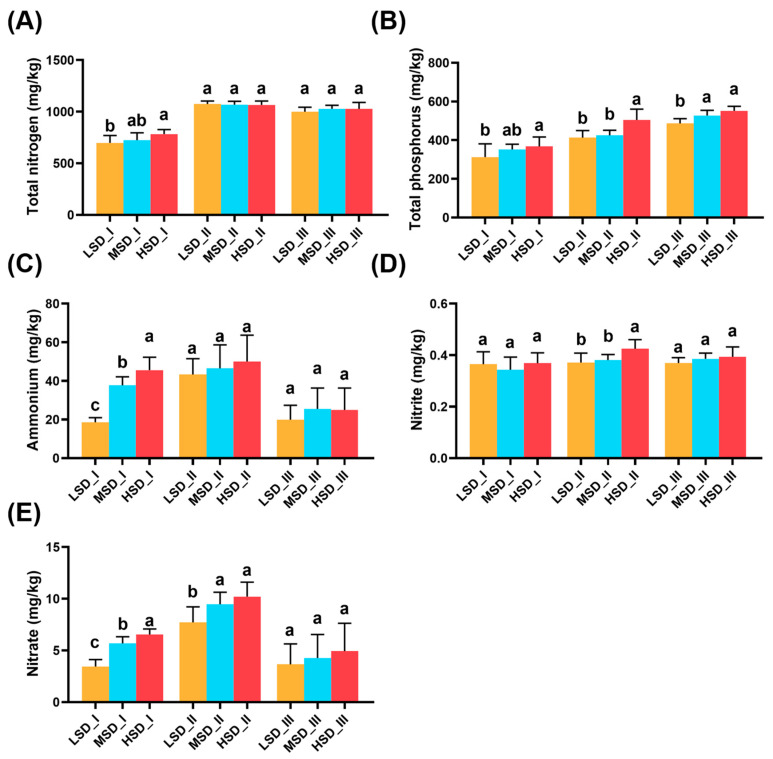
Differences in the environmental factors, including soil TN concentration (**A**), soil TP concentration (**B**), soil ammonium concentration (**C**), soil nitrate concentration (**D**), and soil nitrite concentration (**E**), between the LSD, MSD, and HSD groups throughout the experimental period. The abbreviations LSD, MSD, and HSD corresponded to the low, medium, and high stocking densities, respectively, while the symbols “I”, “II”, and “III” denoted the specific sampling times at culture stages I (30 August), II (30 September), and III (30 October), respectively. The notation of different lowercase letters signified significant differences (*p* < 0.05) among the groups at each sampling time.

Integrated rice-crayfish farming, adopting different stocking densities, significantly affected environmental factors in paddy soil, as illustrated in Figure 7. In culture stage I, TN, TP, ammonia, and nitrate concentrations in the paddy soil increased with stocking density (Figure 7). The HSD group exhibited significantly higher TN and TP concentrations compared with the LSD group, while the ammonia and nitrate concentrations showed significant variation across all groups (Figure 7, *p* < 0.05). No significant differences were noted in nitrite concentration within paddy soil among the LSD, MSD, and HSD groups (Figure 7, *p* > 0.05). During the culture stage II, the TN and ammonia levels did not differ significantly between the LSD, MSD, and HSD groups (Figure 7, *p* > 0.05). However, the soil TP and nitrite concentrations in the HSD group were significantly higher than in other groups, while the LSD group had a significantly lower soil nitrate concentration than that in other groups (Figure 7, *p* < 0.05). By culture stage III, the soil TP concentration in the LSD group was significantly lower than in other groups (Figure 7, *p* < 0.05), with no significant differences in TN, ammonia, nitrate, and nitrite levels across all groups (Figure 7, *p* > 0.05).

**Figure 8:**
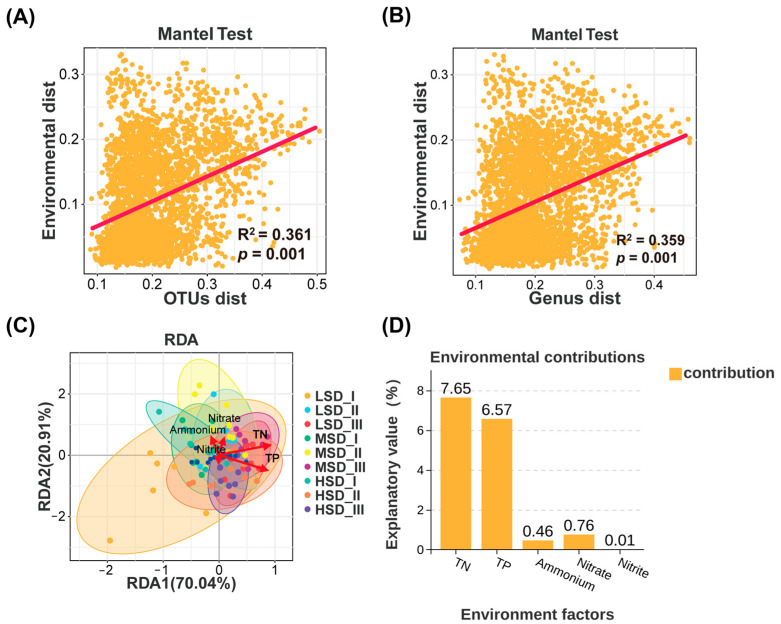
Relationships between the environmental variables and the soil bacterial communities. (**A**) Mantel test to clarify the correlations between the environmental factors and the Operational Taxonomic Units (OTUs). (**B**) Mantel test to elucidate the correlations between the environmental factors and the genus. (**C**) Redundancy analysis (RDA) to highlight the correlations between the soil bacterial community and the environmental factors. (**D**) Envfit test to assess the individual contributions of each environmental factor to the alterations in soil bacterial communities. The abbreviations LSD, MSD, and HSD corresponded to the low, medium, and high stocking densities, respectively, while the symbols “I”, “II”, and “III” denoted the specific sampling times at culture stages I (30 August), II (30 September), and III (30 October), respectively.

Relationships between environmental variables and bacterial communities in paddy soil are illustrated in Figure 8. Mantel test results demonstrated significant associations between the environmental variables and the distribution of both bacterial OTUs (R^2^ = 0.361, *p* < 0.05) and genera (R^2^ = 0.359, *p* < 0.05) in general (Figure 8A,B). According to the RDA, 90.95% of the variations in the structure of bacterial communities within paddy soil could be attributable to environmental factors (Figure 8C). Among all the environmental factors, the TN (R^2^ = 0.340, *p* < 0.05) and TP (R^2^ = 0.367, *p* < 0.05) were obviously correlated with soil bacterial communities, while ammonia nitrogen (R^2^ = 0.049, *p* > 0.05), nitrate (R^2^ = 0.034, *p* > 0.05), and nitrite (R^2^ = 0.005, *p* > 0.05) exhibited no significant correlations. Similarly, the Envfit test also revealed that the TN and TP were the primary contributors to changes in soil bacterial communities, with contributions of 7.65 and 6.57, respectively, greatly higher than those of other environmental factors (Figure 8D).

## 3. Discussion

Our study highlighted the significant impacts derived from varying stocking densities of the red claw crayfish in the integrated rice-crayfish farming systems on the diversities and compositions of soil bacterial communities. Specifically, the high stocking densities of red claw crayfish in integrated rice-crayfish farming could significantly lower the Simpson and Pielou_J indices during the mid-phase of cultivation, indicating reduced diversity and evenness among soil bacteria [43,44]. This effect, however, gradually diminished towards the end of the cultivation period. Additionally, low stocking densities of red claw crayfish initially increased the relative abundance of the phylum *Actinobacteria* in the paddy soil, whereas high stocking densities notably did so during the mid- and final phases. Known for their abundance in soil and crucial ecological functions, *Actinobacteria* play a vital role in breaking down complex organic matter, recycling substances, forming soil aggregates, detoxifying xenobiotics, and stabilizing ecosystems [45,46,47,48]. Meanwhile, *Actinobacteria* harbors numerous plant growth-promoting bacteria and plays a key role in protecting plants from abiotic stresses [49]. Hence, the notable increases in Actinobacteria at varying cultivation stages suggested that low and high stocking densities of red claw crayfish could improve the soil bacterial capacity for organic matter degradation and material cycling and protect plants from abiotic stresses at specific cultivation periods, which might be beneficial for rice growth.

As for the variations in the functional groups for the soil bacterial community, we observed that the integrated rice-crayfish farming with a low stocking density of red claw crayfish initially enhanced the functional groups chemoheterotrophy and aerobic chemoheterotrophy. Conversely, high stocking density promoted the relative abundances of these functional groups during the middle and final culture stages. A high level of aerobic chemoheterotrophy typically signifies numerous bacteria that derive energy from the aerobic oxidation of organic matter [50,51]. Chemoheterotrophic bacteria such as *Proteobacteria* and *Actinobacteria*, functioning as decomposers, are crucial for the restoration environment, recycling of organic material, and contributing to the ecosystem’s carbon cycle [50,51]. The notable increase in functional group chemoheterotrophy and aerobic chemoheterotrophy suggested that at the initial culture stage, the low stocking density of red claw crayfish in the integrated rice-crayfish farming boosted the soil bacteria’s ability to degrade and utilize organic matter. Meanwhile, high stocking density enhanced these processes in the cultivation’s middle and late phases. These results also well corroborated the changes in bacterial community composition within paddy soil discussed above.

Bacterial co-occurrence network patterns significantly differed across paddy soils with varying stocking densities of red claw crayfish in integrated rice-crayfish farming systems. Bacterial co-occurrence networks shed light on the intricate interactions within soil bacterial communities, which cannot be fully captured by simple diversity metrics [52]. Notably, the medium stocking density of red claw crayfish in the integrated rice-crayfish farming system resulted in a more complex bacterial community during the mid- and final culture stages, as evidenced by a higher number of nodes and edges compared to both low and high densities [42]. Such complexity and enhanced connectivity reflect shared ecological traits and an increase in functional redundancy among the bacteria, which can help bacterial communities withstand external pressures and remain stable in the face of environmental change [53,54]. Therefore, our findings indicated that a medium stocking density of red claw crayfish fostered a more complex and stable soil bacterial community in the integrated rice-crayfish farming system [55,56,57].

Previous studies have established that the cultivation and bioturbation of aquatic animals, the introduction of artificial nutrients, and daily management practices can significantly alter environmental factors, thereby affecting environmental bacterial communities [6,58,59,60,61,62,63]. Different stocking densities of the red claw crayfish in the integrated rice-crayfish farming systems led to differences in the amounts of the artificial nutrient inputs and the extent of disturbance from aquatic animals, inevitably resulting in distinct impacts on the environmental factors of paddy soils. Our findings indicated that, during the initial and middle phases of cultivation, the high stocking density of the red claw crayfish induced significantly higher nutrient content in paddy soil compared to the low stocking density or both the low and medium stocking densities, which could be attributed to the comprehensive effects of the artificial nutrient inputs and aquatic animal activities or bioturbation.

Furthermore, strong correlations between environmental factors and soil bacterial communities were revealed in our study. Nutrient resources, particularly TN and TP, play a pivotal role in shaping the soil bacterial community, as they affect the bacterial growth, abundance, and activity [64,65]. Previous studies reported significant correlations between soil bacterial communities and soil TN and TP concentrations [66,67,68,69,70]. Notably, such correlations were also found in various rice production patterns, including integrated rice-fish farming [2,71,72]. Wei et al. [72] observed significant correlations between sediment bacterial communities and both TN and TP concentrations in integrated rice-crayfish farming systems. Arunrat et al. [71] reported a significant relationship between soil TN concentration and soil bacterial community composition in two plant-crayfish coculture ecosystems. Our results well corroborated these studies above, finding that TN and TP concentrations in paddy soil were obviously correlated with the soil bacterial community and emerged as the primary and key environmental factors contributing to the changes in the soil bacterial communities. Hence, variations in the bacterial communities induced by integrated rice-crayfish farming with different stocking densities were predominantly driven by soil TN and TP concentrations.

## 4. Materials and Methods

### 4.1. Experiment Design and Sample Collection

**Figure 9:**
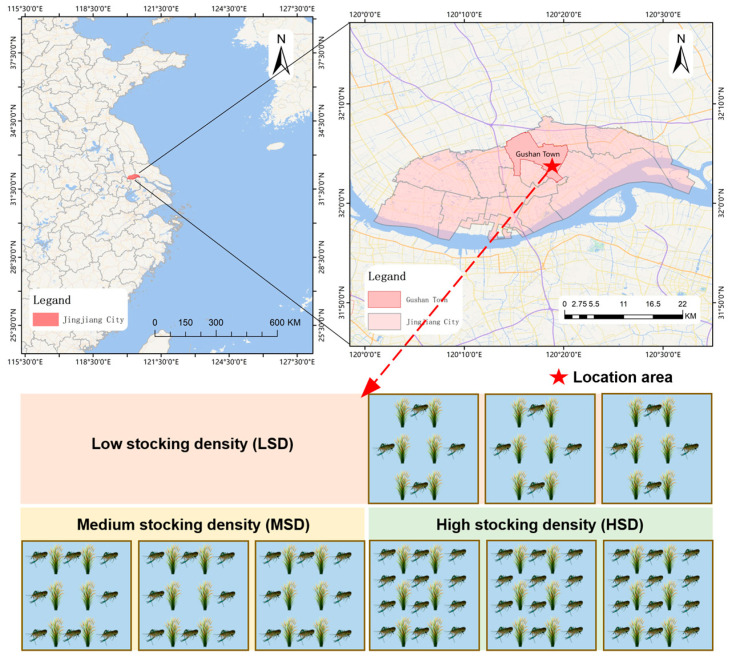
Location of the study area and schematic diagram of the conducted experiment.

This study was carried out at the Jingjiang Experimental Base, Freshwater Fisheries Research Center, located in Jingjiang City, Jiangsu Province, China, with geographic coordinates of E 120°19′56.8″, N 32°5′30.9″ (Figure 9). We utilized nine paddy fields, each covering approximately 400 square meters, for integrated rice-crayfish farming. Three stocking densities were established: low (LSD, 35.73 g·m^−2^), medium (MSD, 71.46 g·m^−2^), and high (HSD, 107.19 g·m^−2^), with each density replicated three times. The rice variety Nangeng 5055 was transplanted into all fields in mid-June 2021 and harvested in early November. On 22 July, approximately 18,000 healthy red claw crayfish (*Cherax quadricarinatus*), averaging 14.29 ± 1.05 g, were introduced into the fields at the specified densities. Rice cultivation and crayfish management across the LSD, MSD, and HSD groups were consistent, adhering to conventional local agricultural practices. Crayfish were fed daily with a commercial diet from Zhejiang Haida Feed Co., Ltd. (Shaoxing, China), containing at least 30% crude protein, 3% crude fat, less than 8% crude fiber, less than 18% crude ash, over 1% total phosphorus, and 1% to 3.5% calcium, based on 2% of their body weight. Water quality parameters, including temperature (23.4–32.1 °C) and dissolved oxygen (3.21–5.08 mg·L^−1^), were maintained within optimal ranges throughout the 90-day experiment.

The paddy soil samples were collected on 30 August, 30 September, and 30 October, corresponding to initial, middle, and final phases of the cultivation period (represented as culture stages I, II, and III), respectively. For each paddy field, we employed the five-point sampling method to collect five paddy soil samples, which were thoroughly mixed to create a single composite sample. This process was conducted three times for each field, yielding three composite samples from each field. Each stocking density group consisted of three paddy fields. Consequently, for each group, we totally collected 45 soil samples and then obtained nine composite samples, and we treated the nine composite samples as nine replicates for subsequent analyses. Following collection, the samples were immediately placed in self-sealing bags and stored at −80 °C for the analysis of ammonium, nitrate, nitrite, total nitrogen (TN), total phosphorus (TP), and bacterial communities.

### 4.2. Environmental Factor Measurements

Paddy soil samples were freeze-dried using a Haier biomedical lyophilizer (model DG-65Z04-10AR, Haier, Qingdao, China) at −50 °C for 72 h. Subsequently, they were ground in a mortar and sifted through a 100-mesh sieve to ensure uniform particle size. The determinations of TN and TP concentrations in paddy soil were performed using the modified Kjeldahl method and the alkali fusion-molybdenum antimony (Mo-Sb) anti-spectrophotometric method, in accordance with Chinese national standards HJ 717-2014 and HJ 632-2011 [73,74], respectively. Moreover, the concentrations of ammonia, nitrate, and nitrite in the soil samples were measured using spectrophotometric methods after extraction with potassium chloride solution, following the protocol outlined in the Chinese national standard HJ 634-2012 [75].

### 4.3. Bacterial DNA Extraction, 16S rRNA Sequencing, and Data Processing

The total bacterial DNA was extracted from the soil samples (27 composite samples in total, with 9 replicates per group) using the HiPure Soil DNA Kits (Magen, Guangzhou, China). For each extracted DNA, we amplified the V3-V4 region of the ribosomal RNA gene with PCR primers 341F and 806R [76]. The PCR reagents were obtained from New England Biolabs, MA, USA. Following amplification, we extracted, purified, and quantified the amplicons with 2% agarose gels, the AxyPrep DNA Gel Extraction Kit (Axygen Biosciences, Union City, CA, USA), and the ABI StepOnePlus Real-Time PCR System (Life Technologies, Foster City, SF, USA). Subsequently, the amplicons were pooled at equimolar concentrations and sequenced on the Illumina platform, utilizing the paired-end 250 (PE250) sequencing method in accordance with standard procedures.

Raw reads were initially filtered using FASTP (version 0.18.0), followed by the merging of clean paired-end reads into raw tags using FLASH (version 1.2.11) [77,78]. Raw tags were then refined to generate high-quality clean tags and clustered into operational taxonomic units (OTUs) based on a minimum similarity threshold of 97% with the UPARSE (version 9.2.64) pipeline [79,80]. Chimeric tags were identified and discarded using the UCHIME algorithm [81]. For each cluster, the most prevalent tag sequence was designated as the representative OTU sequence. These sequences underwent classification using the RDP classifier (version 2.2) against the SILVA database (version 138.1), applying a confidence threshold of 0.8 to ensure accurate taxonomy [82,83]. Finally, OTU abundance data were normalized based on the sample with the lowest tag count, facilitating a consistent basis for further analysis.

### 4.4. Statistical Analysis

Bacterial community alpha diversity indices based on all the OTUs, such as Shannon, Simpson, Chao 1, and Pielou_J, were calculated using QIIME [84]. To examine the variances in soil bacterial communities among the LSD, MSD, and HSD groups, principal component analysis (PCA) based on weighted Bray-Curtis distances, along with the Adonis test, was employed. Circular layout diagrams depicting the composition of the soil bacterial communities were created using Circos (version 0.69-3) [85]. Predictive analysis of the functional attributes of soil bacterial communities within the LSD, MSD, and HSD groups was performed utilizing the Functional Annotation of Prokaryotic Taxa (FAPROTAX) framework [54,86]. To identify co-occurrence patterns and infer potential biological interactions, a co-occurrence network analysis was carried out based on statistically significant correlations (absolute correlation coefficient > 0.6, *p*-value < 0.05) [87]. Additionally, redundancy analysis (RDA), Mantel test, and Envfit test were applied to elucidate the impact of environmental factors on the compositions of the soil bacterial communities. Differences in the environmental variables, bacterial community alpha diversity indices, bacterial community composition, and bacterial community functional groups between the LSD, MSD, and HSD groups were determined through the Tukey’s Honestly Significant Difference (HSD) test.

## 5. Conclusions

For our study, a higher stocking density of red claw crayfish reduced the diversity and evenness of the soil bacterial community during the mid-culture stage. *Proteobacteria, Actinobacteria, Chloroflexi* emerged as the most prevalent phyla throughout the experimental period. Initially, low stocking densities increased the relative abundance of Actinobacteria in the paddy soil, while high densities did so in the later culture stages. There were 90 distinct functional groups identified across all the paddy soil samples, with chemoheterotrophy and aerobic chemoheterotrophy being the most abundant. Low stocking densities initially favored these functional groups, whereas high densities enhanced their relative abundances in the later culture stages. Medium stocking density of red claw crayfish led to a more complex bacterial community during the mid- and final culture stages. Environmental factors showed significant correlations with soil bacterial communities, with TN and TP concentrations emerging as primary factors contributing to the alterations in soil bacterial communities. In summary, our findings demonstrated that integrated rice-crayfish farming with different stocking densities has notably different impacts on the composition, diversity, function, and co-occurrence network patterns of the soil bacterial communities, and soil TN and TP concentrations might be the key factors driving the alterations in bacterial communities under the effects of integrated rice-crayfish farming. The results of this study contribute to theoretical insights into the profound impact of integrated rice-crayfish farming with various stocking densities on bacterial communities in paddy soils.

Our study treated the integrated rice-crayfish farming system as a whole, exploring its impact on soil microbial communities and environmental factors at varying stocking densities. We did not, however, separate the effects of farmed animals’ bioturbation from those of exogenous feed input, which were limitations for our study. Moving forward, we aim to isolate and quantify the impacts of key factors, such as bioturbation by farmed animals and the introduction of exogenous feed on the paddy soil environment. This approach will help us more thoroughly understand how this agricultural production pattern influences the agricultural ecosystem’s underlying mechanisms and principles.

## Data Availability

The bacterial datasets that support the findings of this study are openly available at the National Center for Biotechnology Information (NCBI) with the accession number PRJNA1091544.
